# Printing of Zirconia Parts via Fused Filament Fabrication

**DOI:** 10.3390/ma14195467

**Published:** 2021-09-22

**Authors:** Dorit Nötzel, Ralf Eickhoff, Christoph Pfeifer, Thomas Hanemann

**Affiliations:** 1 Institute for Applied Materials, Karlsruhe Institute of Technology, Hermann-von-Helmholtz-Platz 1, D-76344 Eggenstein-Leopoldshafen, Germany; dorit.noetzel@kit.edu (D.N.); ralf.eickhoff@student.kit.edu (R.E.); 2 Department of Microsystems Engineering, University Freiburg, Georges-Koehler-Allee 102, D-79110 Freiburg, Germany; christoph.pfeifer@imtek.uni-freiburg.de

**Keywords:** fused filament fabrication, 3D printing, FFF/FDM, ceramics, zirconia, material extrusion, MEX

## Abstract

In this work, a process chain for the fabrication of dense zirconia parts will be presented covering the individual steps feedstock compounding, 3D printing via Fused Filament Fabrication (FFF) and thermal postprocessing including debinding and sintering. A special focus was set on the comprehensive rheological characterization of the feedstock systems applying high-pressure capillary and oscillation rheometry. The latter allowed the representation of the flow situation especially in the nozzle of the print head with the occurring low-shear stress. Oscillation rheometry enabled the clarification of the surfactant’s concentration, here stearic acid, or more general, the feedstocks composition influence on the resulting feedstock flow behavior. Finally, dense ceramic parts (best values around 99 % of theory) were realized with structural details smaller than 100 µm.

## 1. Introduction

In recent years, the additive manufacturing (AM) of components made of polymers, metals, ceramics, or composites thereof have attracted many groups worldwide [1,2,3,4,5,6,7,8]. In addition to the different variants of vat photopolymerization, like stereolithography (SLA), material extrusion methods (MEX), especially Fused Filament Fabrication, are widely used for component fabrication beyond commercial material usage. This can be attributed to the relatively simple printer setup and low printer costs and, in the case of the 3D printing of polymer-matrix composites, the exploitation of established techniques from polymer processing, like compounding and shaping. In addition, FFF has been widely investigated, and the impact of the relevant process parameters on the printed device properties is mostly understood [9,10]. Beyond pure polymer printing, polymer-based composites containing inorganic fillers are of particular interest. There are two main material development strategies established. On the one side in the case of polymer-matrix composites (PMC), the addition of an inorganic filler introduces a new functionality like mechanical property reinforcement or dielectric of magnetic properties to the polymer matrix. The aspired impact correlates strongly with the filler amount in the polymer, so highly filled systems are targeted [11]. On the other side, the highly filled polymer is used as a vehicle enabling a certain melt flow for shaping like in powder injection molding (PIM). By convention, in the latter case, the polymer-based composite is called feedstock. After molding, the polymer will be removed, enabling dense ceramic or metal parts [12]. Only a few commercial feedstocks are available for the usage in FFF for the fabrication of metal components [13]. For ceramics, a close cooperation between Fraunhofer IKTS and INMATEC Technologies recently presented the utilization of an alumina feedstock, originally designed for PIM, in FFF [14,15]. Beyond industry, several research groups are engaged in the realization of ceramic and metal components applying the huge bundle of different AM methods [1,4,16,17,18]. In case of MEX, the focus was mainly set on alumina, partially extending the application range to microsystems technology [19,20,21,22,23,24,25]. With respect to ceramic applications, which need enhanced mechanical properties, zirconia components made via MEX should be very promising, but little research can be found in the literature [26,27,28,29]. In 2021, the fabrication of zirconia parts applying MEX methods with an ethylene vinyl acetate containing binder was published [30,31]. A new field of 3D-printing application was opened quite recently, namely the fabrication of lightweight structures considering different materials and printing strategies described, e.g., in [32,33].

The fabrication of ceramic or metal parts via MEX can be adapted from the feedstock development in powder injection molding (PIM). As a common prerequisite, it is strongly recommended to use at least a solid load of 50 vol% ceramic and 60 vol% metal filler to achieve warpage-free and dense sintered parts. This high solid loading is attributed to the sinter process, especially the necessity of a very small particle–particle distance enabling the formation of initial sinter necks at elevated temperatures [12]. Higher solid loadings are favorable; unfortunately, the process-related maximum acceptable feedstock viscosity is exceeded. The biggest difference between PIM and MEX, here FFF, is the occurring shear-rate-dependent viscosity during molding or deposition. Being a high pressure process, the shear rate during molding is significantly higher (~10^3^ 1/s) than in case of the almost pressureless FFF (~10^2^ 1/s). In general, feedstock systems, show a pseudoplastic flow behavior with a pronounced viscosity drop with increasing shear, here equivalent with molding or deposition speed. In addition, the temperature and shear dependent feedstock behavior is in FFF of particular interest when extruded filament contacts the previous printed one. Depending on the viscoelastic behavior, the fresh printed material displaces or distorts the earlier printed material causing a destruction of the aspired device geometry. The temperature and shear dependent viscoelastic properties of a feedstock can be quantified by oscillation measurements enabling the determination of the complex viscosity covering the viscous and elastic portions as well as the material yield point, which is relevant for the previously described item.

In this paper, the modification of the established LDPE and wax binder system to print ZrO_2_ parts successfully via FFF will be presented. In addition to standard feedstock characterization methods, the focus is set on comprehensive oscillation rheometric measurements, gaining a deeper understanding of the viscoelastic behavior of different feedstock compositions. Until now, there have been very few publications of highly filled thermoplastic polymers applying oscillation measurements with respect to use materials in FFF [31,34]. For oscillatory measurements, usually rotational rheometers were used, while the measuring element is not rotating but oscillating with a deformation γ (amplitude) and an angular frequency ω = 2π*f* (period). The deformation induces stress in the three-dimensional network of the feedstocks, which consequently causes cracks upon increasing amplitudes. A further deformation elevation results in a breakdown of the network and the material starts to flow. Due to this, it is recommended to measure shear strain-controlled deformation [35]. For highly filled polymers also, strain-controlled measurements can be found [34], where a strain *τ* (raw data: torque *M(t)*) is given, while the deformation γ (raw data: deflection angle *φ(t)*) and the phase shift angle *δ* is measured. The phase shift angle shows the delay of the material movement answer of the measurement elements stimulation. The deformation of ideal elastic materials like steel starts as soon as torque (more rather strain) is applied, which is why the phase shift angle is 0°. Ideal viscous materials like water have a phase shift angle of 90°; highly filled polymers have a viscoelastic character with a phase shift angle between 0° and 90°. With this given and the measured parameters, the storage modulus G′ and the loss modulus G′′ can be calculated.

$$G^{\prime }=\;\left(\tau /\gamma \right)\cdot \cos\delta$$

$$G^{\prime \prime }=\;\left(\tau /\gamma \right)\cdot \sin\delta$$

The storage modulus G′ represents the stored energy while shearing. As soon as the strain drops, this energy is available and the driving force to move the material towards the origin position [36]. The loss modulus G′′ represents the energy that dissipates during shearing, for example by temperature increase due to friction processes [34,36,37].

## 2. Materials and Methods

With respect to the realization of dense zirconia parts applying FFF, a process chain was adapted from ceramic injection molding. This approach was successfully demonstrated earlier, applying two different binder systems like wax/polyethylene [23] and polyethyleneglycol/polyvinylbutyral [24,25] for the realization of dense sintered alumina parts. The process development covers the following aspects listed below, but it has to be stressed that each individual step has to be evaluated carefully including a comprehensive material characterization in order to develop a robust process chain allowing dense and warpage-free zirconia parts:

1. Suitable material (filler, binder, surfactant) selection
2. Compounding and flow properties characterization
3. Filament extrusion
4. Printing via FFF
5. Debinding and sintering.

### 2.1. Material Properties and Feedstock Composition

As presented in earlier work dealing with reaction or micro-powder injection molding [38,39,40,41] a submicron sized zirconia (ZrO_2_, TZ-3YS-E, Tosoh, Tokyo, Japan) was selected as ceramic material. The relevant particle properties were measured as follows:

- Particle size distribution: Laser diffraction (LA-950 Horiba Ltd., Kyoto, Japan)
- Specific surface area: BET method (Gemini VII 2390, Micromeritics Instruments Corp., Norcross, GA, USA)
- Particle density: Helium pycnometry (Pycnomatic ATC, Porotec, Germany)
- Particle morphology: SEM (Supra 55, Zeiss).

The polymer binder consists mainly of two components. A low molecular weight paraffin wax (Sasolwax 6403, Sasol Wax GmbH, Hamburg, Germany), with a melting temperature of 60–65 °C, enables a low melt viscosity at moderate temperatures, and a Low Density Polyethylene (LDPE, Lupolen PE1800H, LyondellBasell, Frankfurt, Germany) having a melting temperature around 110 °C guarantees a certain mechanical stability at low temperatures. Stearic acid (SA, Roth GmbH, Karlsruhe, Germany) has been selected as surfactant with superior properties increasing the compatibility between the polar ceramic and the nonpolar thermoplastic enabling a low feedstock viscosity. To ensure high material densities after sintering, the solid content in all feedstocks is set to 50 vol%. This value is a compromise between getting a moderate feedstock viscosity and acceptable sinter shrinkage [12].

In previous work, the volume ratio of the binder components (LDPE and wax) was fixed to 50:50. However, first results showed that even the viscosity of the material with the highest SA loading was too high to ensure satisfying FFF printing results. Therefore, another compound with a ratio of LDPE:wax was investigated.

### 2.2. Compounding and Feedstock Characterization

All zirconia-based feedstocks were prepared in a mixer–kneader compounder (W50-EHT, Brabender, Duisburg Germany) with simultaneous torque recording during mixing with a constant blade rotation of 30 rpm. The compounding temperature was set to 125 °C, while a fixed mixing time of 60 min guaranteed the formation of a homogeneous feedstock. It is important that in all cases, the same sequence of material addition into the mixing chamber (volume 45 mL) is kept, a detailed description is given in [24]. The melt viscosity of all feedstocks was measured with a high-pressure capillary rheometer (Rheograph 25, Göttfert, Buchen, Germany) at a temperature of 160 °C, a capillary diameter 1 mm and a capillary length 30 mm. The shear rate was varied in the range from 0.5 until 15,000 1/s. The apparent values of shear rate, stress and viscosity were corrected following the Rabinowitch-Weissenberg approach. The viscoelastic properties have been measured by oscillation rheometry applying a Malvern/Netzsch Gemini HR Nano (Herrenberg, Germany) at 160 °C with the geometry of the measuring system PP-20 and a gap of 2 mm. With respect to the intended application of the feedstocks in a FFF-printer, where the deformation cannot be adjusted, the shear stress and its direct impact on the viscoelastic behavior were controlled. The amplitude sweep (AS) had been shear stress controlled at 1 Hz (ω = 6.28 rad/s). The strain in frequency sweep (FS) was carried out at 100 or 400 Pa, depending on the mixing ratio of LDPE to wax.

### 2.3. Filament Extrusion and Printing

The pelletized feedstock obtained from the rheological investigation were shaped into filaments applying a one-screw filament extruder (Noztek pro HT, Noztek, Shoreham, UK) at a temperature of 160 °C. The nozzle diameter was set to 2.8 mm to meet the requirements of the modified print head of the employed German RepRap X350pro (Feldkirchen, Germany) FFF printer. As in case of the related alumina-based feedstock, the stiff and rigid filament cannot be winded and was cut every 50 cm for a direct feeding into the print head. The pristine factory-made print head was modified according to the filament diameter of 2.85 mm and conveying enhancing the accuracy of the volume amount of extruded material for better surface quality of the printed samples. A more detailed description can be found in [23,24]. Simplify3D and Cura (Ultimaker BV) were used as slicing software.

### 2.4. Thermal Postprocessing: Debinding and Sintering

It has been shown earlier that prior to thermal debinding, a chemical debinding helps to retain the printed shape of the device [42,43]. In this first step, the paraffin wax is solved in n-hexane, while the LDPE remains in the sample. This pretreatment opens channels in which the gaseous decomposition products like water, CO_2_ and molecular fragments can leave the printed part. In addition, a thermal debinding (HT5/28, Carbolite, Neuhausen, Germany) up to a temperature of 500 °C with small heating rates followed for complete removal of all organic moieties. Finally, sintering occurred in a chamber oven (RHF17/3, Carbolite, Neuhausen, Germany) at a temperature of 1450 °C. The Archimedes method using a Sartorius balance was applied for the sample density measurement. The geometric shrinkage after sintering was measured using a Heidenhain CT60M measuring system (14 test specimen: solid load 50 vol% ZrO_2_, binder composition: 40 % LDPE, 60 % wax). Microscopic images were taken with a Leitz Orthoplan (Wetzlar, Germany) microscope equipped with RSF Elektronik Z520 (Tarsdorf, Austria; x,y-directions) and Heidenhain MT25 (z-direction, Traunreut, Germany) length measuring systems. The surface profiles were measured applying a white light interferometer (Micro Prof^®^ 100, Fries Research Technology, Bergisch Gladbach, Germany). The sample micrographs (cross-section) were prepared applying the following steps:

- Grinding with different diamond grinding wheels (70 µm until planarity, 40 µm for 30 s, 10 µm for 2 min)
- Polishing with diamond paste (6 µm, 3 µm) and lubricant, each 30 min, surface pressure 25 N, wheel rotation speed 150 rpm.

## 3. Results and Discussion

### 3.1. Material Properties

**Figure 1:**
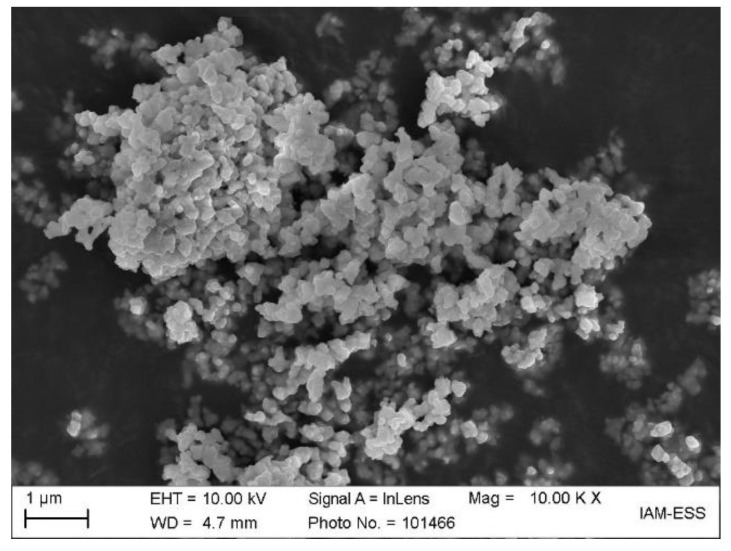
SEM-image of the used zirconia TZ-3YS-E showing the particles morphology.

In Table 1, the measured particle properties of the selected zirconia TZ-3YS-E are depicted. According to the particle size distribution, the d_50_-value is around one µm enabling a moderate sinter temperature around 1450 °C following the vendor’s recommendation. The measured specific surface shows a value around 6.6 m^2^/g, which is significantly smaller than the previously used alumina [23,24]. The specific surface area is important for the calculation of the necessary surfactant amount. SEM-investigations reveal a highly aggregated ceramic with primary particle sizes of 100–300 nm, where both soft and hard agglomerates can be seen (Figure 1). Helium pycnometry delivered a density value of 6.01 g/cm^3^, which is a little bit smaller than the value (6.05 g/cm^3^) supplied by the vendor Tosoh.

### 3.2. Compounding and Feedstock Characterization

In the literature, torque recording measurements with variable blade rotational speed can be used to obtain more detailed information in certain cases like polymer blends [44]. Here, the investigated system is more complex, consisting of an organic binder mixture, a surfactant and an inorganic filler. With respect to shaping, the most important point is to achieve a homogenous feedstock, which can be seen from the time-dependent torque progress at constant rotational speed. In addition, the influence of surfactants and their concentration on the flow behavior is relevant for feedstock development [45].

**Figure 2:**
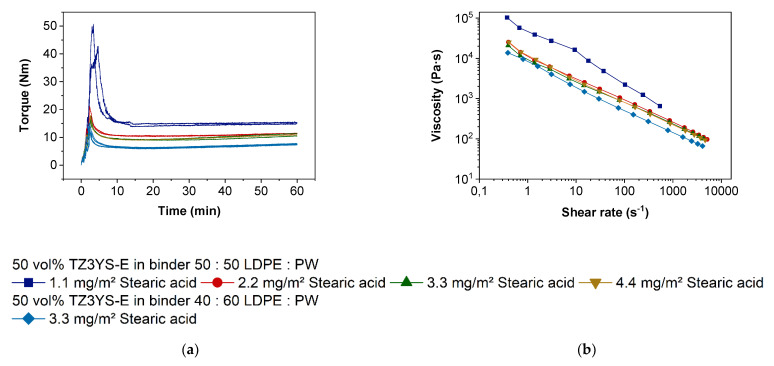
Characterization of the feedstocks flow properties as function of the stearic acid concentration: (**a**) Time dependent torque evolution during compounding (T = 125 °C); (**b**) Shear rate dependent melt viscosity (T = 160 °C).

With respect to a homogenous feedstock composition, the compounding procedure has to be performed very carefully and standardized. It is known from previous work dealing with feedstock development for FFF or injection molding that a compromise for the surfactant concentration has to be found, which ranges normally between 1.1 and 4.4 mg surfactant/m^2^ filler specific surface area. The amount of SA to achieve a surfactant monolayer on a particle surface was calculated to be around 2.27 mg/m^2^ specific surface area [46]. The compounding process can be split into three main states. First, the filling state with the addition of all individual components in a predefined order; second, the mixing state, where the surfactant and the other organic compounds start wetting the filler particles; and finally, the equilibrium state where all agglomerates should be disrupted and the particles are completely wetted in ideal circumstances [23]. The respective recorded torque vs. time diagram for the variation of stearic acid amount is displayed in Figure 2a. The feedstock with the smallest stearic acid concentration shows the highest torque, both during the initial mixing state and the final equilibrium state after one hour. A pronounced torque drop can be observed at higher surfactant amounts, but there is no remarkable difference between 2.2 and 4.4 mg/m^2^. The lower concentrations were investigated twice to prove the reproducibility of the compounding process. A similar behavior can be observed during the measurement of the feedstock´s melt viscosity at 160 °C (Figure 2b). This is comparable to the results of Tseng et al., who observed no decrease in viscosities lower than 1.0 m^2^/g zirconia [47]. They observed agglomerates in the green bodies, which lead to a change of suspension structure and low green part densities. SA amounts of 1.6 mg/m^2^ decrease viscosity and even more 2.6 mg/m^2^. From the obtained results, it can be derived that starting with a 2.2 mg/m^2^ stearic acid concentration, a complete wetting of the zirconia’s surface is achieved. This delivers a pronounced viscosity drop up to a factor of 10 at lower and moderate shear rates, which are relevant for the FFF printing process. Measurements of Auscher et al. showed that using a SA content in feedstocks > 3.1 mg/m^2^ of zirconia with a specific surface of 6.7 m^2^/g, which is very compatible to the zirconia utilized in this paper, lead to a saturation of the powder surface at 1.6 mg/m^2^ surfactant concentration [48]. It can be expected, that only amounts > 3.1 mg/m^2^ SA cover the surface statistically. By experience and as a compromise it is recommended to apply a stearic acid content of at least 2.2 or 3.3 mg/m^2^. It was observed in injection molding experiments that very large surfactant amounts lower the stability of the green body. With respect to a reduction in the feedstock’s viscosity and retaining the solid content constant, the LDPE amount was decreased, and the wax content increased. It can be seen in both Figure 2a,b that mixing torque and viscosity decrease with increasing wax and SA content, which is comparable to the results of the viscosity measurements of alumina in the same binder system [49]. According to shaping, FFF or injection molding, it is mandatory that a constant torque value is reached after a certain compounding time guaranteeing a homogenous feedstock quality (Figure 2a).

Figure 2b shows a strong shear thinning behavior, which is generally expected for highly filled polymeric materials. To investigate the influence of SA on the rheology in more detail, oscillatory measurements were done. During the sample preparation for the oscillation measurements, it was recognized that the feedstock with the amount of SA = 1.1 mg/m^2^ was not flexible enough at the measuring temperature to be squeezed into the gap of 1 mm. The pressure to close the gap down to 1 mm had been too high for manual sample preparation. Therefore, and for better compatibility, all materials were measured at a gap of 2 mm.

Amplitude sweeps (AS) are commonly used for measuring the length of the linear viscoelastic range (LVE), where the deformation of a material is completely reversible. However, amplitude sweeps can help us to understand the materials behavior in shaping processes too, especially for 3D-printing via FFF, because the force to extrude the filaments through nozzles is limited [50]. In this oscillatory measurement, two characteristic points of materials amongst others can be defined: the yield and the flow point. At the yield point, the inner three-dimensional network starts to collapse and the feedstock deformation is no longer completely reversible, but G′ is still larger than G′′. The crossover of the storage and the loss moduli (G′ = G′′), at which the material is a viscoelastic fluid in a rheological meaning, is denoted as the flow point [34]. The shear stress level of the flow point is mandatory to move the material for shaping in forming processes.

**Figure 3:**
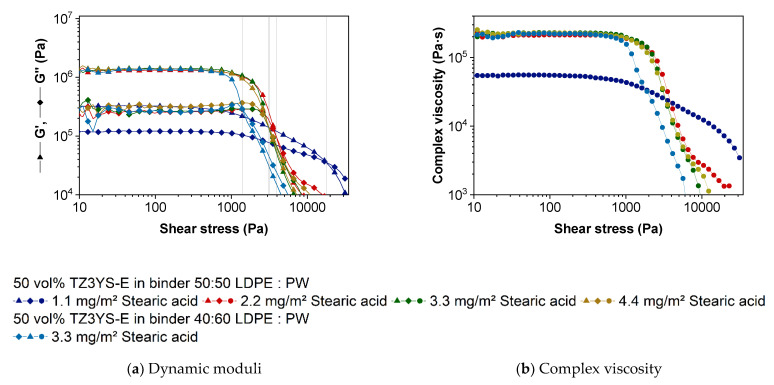
Amplitude sweep measurements of the investigated feedstocks: (**a**) Storage and loss modulus as function of the shear stress; (**b**) Complex viscosity as a function of the shear stress.

In Figure 3a, G′ and G′′ as function of shear stress are displayed. In all cases, the storage modulus is at low shear stresses larger than the loss modulus. Both remain constant with increasing stresses (LVE). By decreasing the stresses within this LVE, the material would reverse to the original position. At higher shear stresses and at the end of the LVE, the three-dimensional network begins to collapse and both moduli decrease at the softening point. Even if G′ is higher than G′′, it decreases at lower shear stresses with a stronger decay slope so that both moduli cross each other, and the loss modulus is dominant. Passing this point G′ = G′′, the feedstocks show the behavior of a viscoelastic liquid. It is shown that all of the investigated feedstocks not only have a shear thinning behavior but also a yield point that is extremely important for 3D-printing via FFF. That means former printed layers could resist the applied pressure by overprinting the subsequent printed layers retaining their position and shape of the built part geometry.

In Figure 3a, the material with 1.1 mg/m^2^ SA shows decreased dynamic moduli that could be usually observed at low solid contents [34,37,51]. Since the solid content are constant in all materials, the SA is likely responsible for the difference in the rheological behavior. On the one hand, the feedstock with 1.1 mg/m^2^ uncovered particle surfaces could have a higher friction while flowing than particles with a monomolecular SA layer. On the other hand, there could remain undestroyed agglomerates or bridging flocculation in the feedstock resulting from an incomplete surface coverage due to a lack of SA [37]. In Figure 3b, the complex viscosity of the different materials is depicted. In contrast to the capillary rheometer measurements, the feedstock with only 1.1 mg/m^2^ SA shows a lower viscosity than the other material, but it must be noticed that the viscosity does not decrease in the same level by leaving the LVE. While being subjugated to high shear stresses, the material with the lowest SA amount reveals higher resistance against the movement equivalent with a higher viscosity. Additionally, the yield point of G′ = G′′ of the feedstock with the lowest amount of SA differs from the other feedstocks with the same binder composition. A significant higher shear stress must be applied to transform this viscoelastic solid to a movable viscoelastic liquid. This is consistent with the observed phenomenon to achieve a small gap prior to the oscillation measurement described earlier.

The results of the oscillation measurements of the feedstocks with 2.2–4.4 mg/m^2^ are very similar. The crossover (grey vertical lines for better visualization) of G′ = G′′ align around 3095–3153 Pa (SA content of 1.1 mg/m^2^ of 17721 Pa), which is in the range of the measurement inaccuracy. Only the loss modulus G′′ increases marginally with increasing SA amount. Because $\tan\delta =G^{\prime \prime }/G^{\prime }$, the phase shift angle increases, and the feedstocks show a more viscous behavior with increasing SA amount. In agreement with the viscosity measurements obtained from a high-pressure capillary rheometer, increasing SA contents show a very strong influence on the resulted feedstock properties. Both G′ and G′′ are higher, and much more importantly, the yield point decreases drastically. That means less strain is necessary to deform these feedstocks irreversible. In the presented system of ZrO_2_-LDPE-PW, an amount of only 2.2 mg/m^2^ SA is necessary to have a strong rheological effect.

The feedstock with the modified binder ratio of LDPE:PW = 40:60 provides only a minimal lower storage modulus but a similar loss modulus like the initial feedstock with a 50:50 ratio. Noticeable is the shorter LVE and the crossover G’ = G’’ that is reached at only 1388 Pa. This material can be deformed irreversible by much less force than the other feedstocks. Due to the lower viscosity at higher shear rates, less force is necessary to extrude the partially molten feedstock especially by using small nozzle diameters. As already mentioned, extruders in common 3D-printers provide limited forces [50]. However, much more important is the trend of the filaments to buckle or shredding [50,52,53], if the resistance to extrude is too high, which can occur besides clogging, but also due to very high viscosities in order of low printing temperatures, high solid contents or inappropriate binder compositions.

Frequency sweep (FS) tests are usually carried out to learn about molecular masses and molecular mass distribution of polymers. In addition, the short- and long-time behavior, e.g., of slurry settlement during storing or transport, can be estimated. The authors believe that in the case of highly filled thermoplastic polymers, the information, which can be received by this kind of measurement, is limited. Stable dispersions are characterized by a strong three-dimensional network due to high interaction forces of particles and molecules, which results in G′ > G′′ in the whole frequency range [36]. Similar to cross-linked polymers, which consist of a three-dimensional network as well, the dynamic moduli are more or less independent of frequency, if measured in LVE [36], and are an indicator for pronounced particle–particle interactions [34,35]. Rueda et al. reported that the storage modulus in almost all highly filled systems is nearly independent of the frequency and increase with solid content [37].

**Figure 4:**
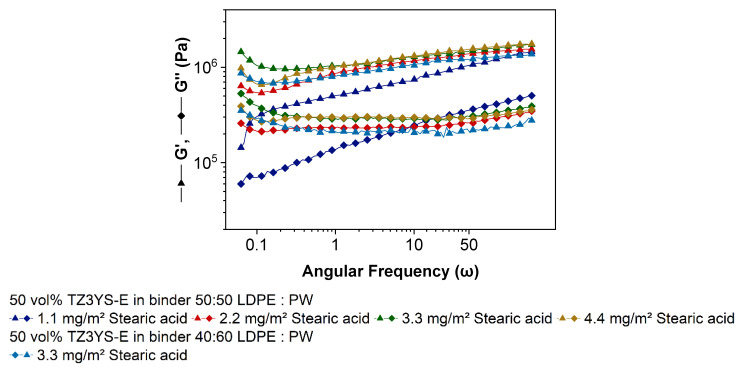
Oscillation measurements, here frequency sweep: Storage and loss modulus as function of the frequency.

In Figure 4, the frequency dependence of the dynamic moduli is shown. The storage modulus dominates the loss modulus over the complete frequency range, while both moduli only very slightly decrease with decreasing frequency. Except the material with a SA content of 1.1 mg/m^2^, all presented curve progressions are very similar to measurements described in [34,35,54,55]. Only the level of G′ and G′′ are varying because of different filler materials, solid loadings and polymers. Auscher et al. reported a strong drop of the storage modulus at a SA content of 2.2 wt% relative to solid weight (equivalent to 3.1 mg/m^2^) [48], which could not be reproduced in our experiments. A high storage modulus indicates high particle–particle attraction forces, which decrease by increasing SA content [48]. That suggests the assumption of low particle interactions in the presented feedstocks with 1.1 mg/m^2^ SA. As described in Figure 4, the material with the lowest SA amount behaves differently than the other feedstocks. Even if G′ > G′′, both moduli decrease parallel with decreasing frequency. That is an indicator that the binder is dominating the viscoelastic properties of the system [34] and is quite unusual for highly filled polymers. Because the binder composition is identical, the observed behavior must be attributed to the concentration of stearic acid.

The rheological results of capillary and oscillation measurements indicate that the feedstock with the smallest SA content of 1.1 mg/m^2^ has got a low viscosity at low and a high viscosity at high shear rates. Because viscosity of powder filled polymers while moving is mainly a surface effect, the powder seems to have a shear rate dependent surface area. The basic function of SA is to support the deagglomeration and the reduction of the particle–particle friction while shearing, but at least a monolayer coverage of the fillers surface by the surfactant is mandatory for fulfilling these tasks. If this is not achieved, the surface and shear-rate-dependent viscosity can be explained. While mixing and measuring in the capillary rheometer, powder agglomerates are destroyed due to appearing shear forces, but the agglomerates can recover due to an insufficient amount of SA that covers the increasing surface during shear-induced deagglomeration. The deagglomeration causes an increase in the fillers surface area which acts as an interface to the binder and other particles. If the agglomerate destruction dominates the agglomerate recovering process, the resulting viscosity is very high. While oscillatory measurements the feedstock showed a low viscosity at low shear rates; hence particle reagglomeration is dominating due to low shear forces, which results in large agglomerates like in systems with large primary particles possessing low viscosity values. Especially in AS measurements by observing the complex viscosity this assumption can be explained. Even if the viscosity of 1.1 mg/m^2^ SA-containing material is lower than all other feedstocks, for deagglomeration, significant higher shear stresses are necessary. At and passing the yield point, the viscosity of the 1.1 mg/m^2^ feedstock does not drop as strong as the others. Primary particles deagglomerate and “new uncovered surface” forms, which results in higher inner friction and higher viscosity. The constant decrease in the dynamic moduli by decreasing frequency in FS indicates unstabilized particles in the matrix.

### 3.3. Filament Extrusion and Printing

As described in previous work [23,24], the diameter of the filament should not vary more than ±0.1 mm. This prerequisite is fulfilled in case of the highly filled filaments [23], where the influence of the viscoelastic polymer with its intrinsic extrudate swelling after passing the extruder nozzle is almost suppressed. Starting from previous work using the LDPE/wax binder system [23], the following FFF printing parameters have been evaluated as comprise regarding for best component quality (bulk, surface, corners):

- Print head extruder temperature: <170 °C
- Printing speed: 10 mm/s
- Platform temperature: 70 °C
- Smallest nozzle diameter: 0.4 mm
- Layer height: 0.1 mm

Some of the above-listed parameters possess a very small process window. If the print head extruder temperature is lowered, the material cannot be extruded or grinding as well as filament fracture occurs. At elevated temperature, wax and SA evaporate or decompose. The platform temperature should be in the range of 60–75 °C; the printing speed should not exceed 10 mm/s. Otherwise, the increased material conveying in the nozzle needs higher pressure causing a filament fracture of grinding. A clogging was not observed within the applied printing parameters.

### 3.4. Thermal Postprocessing: Debinding and Sintering

After treatment with solvent, the printed samples were thermally debinded according to the temperature program listed in Table 2. It is important to apply small heating rates to minimize the generation of internal stress due to thermal expansion coefficient mismatch of the binder and the ceramic, as well as the evolution of gaseous decomposition products and the related significant volume increase. The sinter program is provided in Table 3 with a heating and cooling rate of 5 °C/min between ambient and selected sinter temperature of 1450 °C for maximum density values of max. 6.08 g/cm^3^ for unprinted filaments.

**Figure 5:**
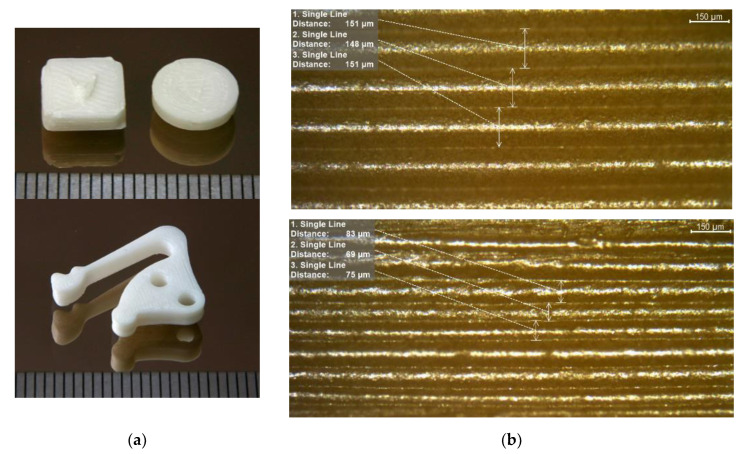
Sintered zirconia samples: (**a**) Test structures for density measurements and lever arm type sample (distance between dark lines at the bottom is 1 mm); (**b**) Measurement of printed layer thickness as smallest geometric feature.

Figure 5a shows two different test specimens, which were used for the sinter part density measurements via Archimedes method, as well as a more complex part with different structural features like boreholes and large cantilever arm. At the bottom of the two images in Figure 5a, one can find a scale bar with 1 mm distance between two dark lines. More test specimen details like geometry can be found in [49]. To estimate the smallest geometric features, which can be realized with the given material composition and printing parameters, a light microscopy image of the narrow side can be derived from Figure 5b. To increase the contrast for light microscopy, the ceramics were coated with graphite. In the upper part layers with a printing height of 200 µm and in the lower part origin layer thicknesses of 100 µm are displayed. Due to sinter shrinkage these layer heights result in 150 ± 7 µm and 78 ± 8 µm, measured at >10 layers. Considering the uncertainty coming from the optical measurement, the layer thickness can be assumed as almost constant and being smaller than 100 µm in the latter case. A closer look into the image section partially shows a wavy structure of the sintered layers, which has its origin in the printing process.

**Figure 6:**
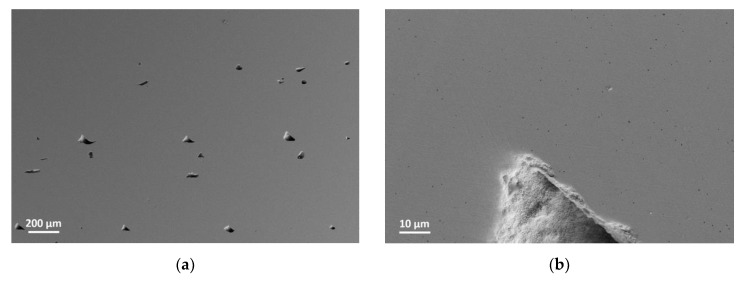
Micrographs of a sintered zirconia sample at different magnifications: (**a**) Voids; (**b**) Pores.

**Figure 7:**
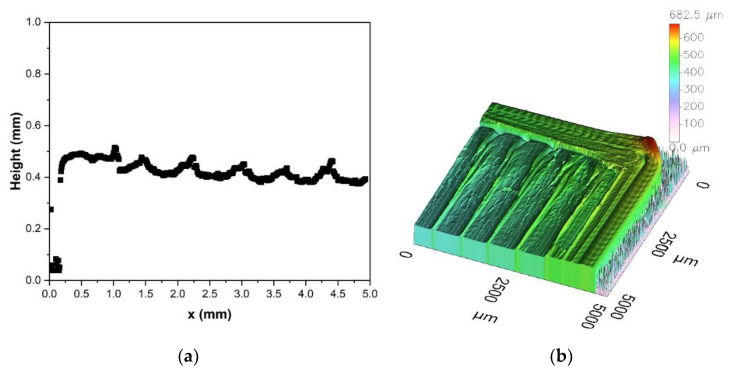
Surface profile of a printed greenbody: (**a**) 2D-scan perpendicular to printing direction; (**b**) 3D-scan.

The micrograph of a sintered disc with 99 % of sintered filaments density is shown in Figure 6. The process-related triangular voids (Figure 6a) are not as periodic as commonly known because the printing direction turned about 45° with each layer. Thus, every fifth layer, the pattern should be similar. The samples were printed with an infill of 100 %, which is usually not enough to fill the printing process-related voids. However excess material due to deviations of the filament diameters or not exact distances between the deposited filament traces because of stepper motors moving inaccuracies can fill the voids partly. Nevertheless, the huge measured bulk sinter density of 99.2 % Th. confirms satisfactory filled voids. In Figure 6b pores in comparison to the process-related voids are depicted. The image indicates a very fine grain size. Moreover, the lack of powder nests shows a good deagglomeration and wetting during compounding as well as a successful sinter step. The surface quality of a green zirconia part is presented in Figure 7. From the line scan (Figure 7a), perpendicular to the printing direction as well from the 3D surface scan (Figure 7b), the pristine shape of the deposited filaments can be recognized. The individual printed threads are closed packed without any visible defects like wholes.

### 3.5. Process Validation

With respect to a validation of the presented process chain, Table 4 summarizes the main results. The listed data shows the closeness of FFF to the powder injection molding process chain. In principle, the obtained results are close to the literature values for zirconia parts [40], especially the maximum achievable ceramic part density. In contrast to the given results obtained by FFF of zirconia feedstocks, injection molding enabling almost defect-free samples without inner voids allowing for excellent mechanical properties. The measurement of the x,y,z-geometry shows an almost isotropic shrinkage. As a drawback, PIM always needs a mold, which can be, depending on the surface structure, very expensive, especially if structural features below 100 µm are targeted. If reduced mechanical properties are acceptable depending on the aspired application, FFF allows more design freedom and a rapid prototyping of zirconia parts, which can be seen, e.g., at the cantilever structure (bottom of Figure 5a).

## 4. Conclusions and Outlook

In this work, a process chain for the realization of sintered zirconia parts containing the individual steps compounding, FFF-printing, debinding and sintering was developed. A more detailed investigation of the rheological behavior of the different feedstock systems applying oscillation rheology delivered a better understanding of the stearic acid influence on the flow behavior especially at low surfactant concentration and low shear stress, which represents the flow situation at the extruder nozzle of the FFF print head. It was possible to establish a feedstock composition with 50 vol% zirconia loaded with low melt viscosity values suitable for FFF printing. After component printing, a combination of solvent pre-treatment and thermal debinding as well as sintering enabled dense ceramic parts with excellent sinter density values around 99 % Th. Future work could focus on the one hand on feedstock composition with higher ceramic load and on the other hand on the printing of more complex parts and finer structural features as well as on the further reduction of voids allowing sintered parts with good mechanical properties.

## Figures and Tables

**Table 1:** Properties of the used zirconiaTZ-3YS-E.

Vendor	Grade	d_10_ (µm)	d_50_ (µm)	d_90_ (µm)	Specific Surface Area (m²/g)	Density (g/cm³)
Tosoh	TZ-3YS-E	0.34	1.04	2.85	6.6	6.01

**Table 2:** Thermal debinding parameters.

Step/Temperature (°C)	Rate (°C/min)	Dwell Time @ Temperature (min)
RT → 120	0.2	120
180	0.2	120
250	0.2	120
500	0.2	60
RT	n.a.	n.a.

**Table 3:** Sinter program.

Step/Temperature (°C)	Rate (°C/min)	Dwell Time @ Temperature (min)
25–1450	5	180
1450–25	5	n.a.

**Table 4:** Collection of geometric parts properties.

Feature	Zirconia
Maximum solid load of FFF-printable feedstock	50 vol%
Average density of sintered parts	5.96 ± 0.11 g/cm^3^
Average shrinkage in x,y,z directions	x: 20.7%; y: 20.9%; z: 21.3%
Max. ceramic part density	99.2% Th
Average smallest structural detail (z-axes)	78 ± 8 µm

## Data Availability

Not applicable.
